# Influence of Residue and Nitrogen Fertilizer Additions on Carbon Mineralization in Soils with Different Texture and Cropping Histories

**DOI:** 10.1371/journal.pone.0103720

**Published:** 2014-07-31

**Authors:** Xianni Chen, Xudong Wang, Matt Liebman, Michel Cavigelli, Michelle Wander

**Affiliations:** 1 College of Resources and Environment, Northwest A&F University, Yangling, Shaanxi, PR China; 2 Department of Agronomy, Agronomy Hall, Iowa State University, Ames, Iowa, United States of America; 3 Sustainable Agricultural Systems Lab, Agricultural Research Center, Beltsville, Maryland, United States of America; 4 Department of NRES, University of Illinois, Urbana-Champaign, Illinois, United States of America; Agricultural Research Service, United States of America

## Abstract

To improve our ability to predict SOC mineralization response to residue and N additions in soils with different inherent and dynamic organic matter properties, a 330-day incubation was conducted using samples from two long-term experiments (clay loam Mollisols in Iowa [IAsoil] and silt loam Ultisols in Maryland [MDsoil]) comparing conventional grain systems (Conv) amended with inorganic fertilizers with 3 yr (Med) and longer (Long), more diverse cropping systems amended with manure. A double exponential model was used to estimate the size (*C*_a_, *C*_s_) and decay rates (*k*_a_, *k*_s_) of active and slow C pools which we compared with total particulate organic matter (POM) and occluded-POM (OPOM). The high-SOC IAsoil containing highly active smectite clays maintained smaller labile pools and higher decay rates than the low-SOC MDsoil containing semi-active kaolinitic clays. Net SOC loss was greater (2.6 g kg^−1^; 8.6%) from the IAsoil than the MDsoil (0.9 g kg^−1^, 6.3%); fractions and coefficients suggest losses were principally from IAsoil’s resistant pool. Cropping history did not alter SOC pool size or decay rates in IAsoil where rotation-based differences in OPOM-C were small. In MDsoil, use of diversified rotations and manure increased *k*_a_ by 32% and *k*_s_ by 46% compared to Conv; differences mirrored in POM- and OPOM-C contents. Residue addition prompted greater increases in *C*_a_ (340% vs 230%) and *C*_s_ (38% vs 21%) and decreases in *k*_a_ (58% vs 9%) in IAsoil than MDsoil. Reduced losses of SOC from residue-amended MDsoil were associated with increased OPOM-C. Nitrogen addition dampened CO_2_-C release. Clay type and C saturation dominated the IAsoil’s response to external inputs and made labile and stable fractions more vulnerable to decay. Trends in OPOM suggest aggregate protection influences C turnover in the low active MDsoil. Clay charge and OPOM-C contents were better predictors of soil C dynamics than clay or POM-C contents.

## Introduction

To manage soil sustainability, crop rotation, tillage, fertilization and other management practices must be combined in ways that improve or maintain soil carbon stocks and reduce net carbon loss [1]–[3]. Use of diversified farming systems that rely on crop rotation and return of crop residues and/or manures is advocated as one way to improve agricultural sustainability and increase soil organic carbon (SOC) [4]–[6]. Diversified crop rotations benefit the soil by varying the quantity, quality, and spatial and temporal placement of organic matter inputs, which therefore altered the physical and biochemical factors and influenced decay of SOC [7]. The ability of the management practice to alter SOC is likely to vary with inherent soil properties, such as clay content, mineralogy and pH [8]–[10]. Dynamic properties like particulate organic matter (POM) that are sensitive to management and change within relatively short time frames (years to decades) may help us predict whether or how SOM status might be improved. Failure to accumulate SOC with increased C additions can occur when soil is already C saturated [11], or in instances where decay rates are high due to stoichiometric imbalance usually caused by high levels of available N [12], [13]. Several studies suggest that manure application can accelerate SOC decay rates [14], [15]. Both the frequency of manure addition and crop rotation length (diversity) have been linked to increased levels of available soil N [16]. This suggests that longer rotations with less frequent manure application might be better able to sequester C in soil. In order to optimize soil C cycling to maintain soil productivity and environmental function, we need to be able to predict how soils C dynamics will respond to management.

Soil incubations provide one method to study mechanisms controlling soil C turnover under variable amendment regimes. Incubation data are often divided into ‘active’ (*C*_a_) and ‘slow’ (*C*_s_) carbon pools by fitting CO_2_ release data to a two-pool model [3], [17]–[19]. Estimates of *C*_a_ and *C*_s_ are often compared against direct measures of SOC fractions, such as total POM and occluded-POM or microbial activity, in an attempt to validate/evaluate models and improve their structure [20], [21]. Density or particle size fractionation may provide a useful way to quantify changes in physical protection and improve our ability to delineate active and slow C pools and understand their dynamics [22]. Incubations provide a way to carry out controlled evaluation of how inherent and dynamic soil properties influence soil response to residue and N additions.

Residue additions can increase C mineralization and positive priming (which is the acceleration of native SOC decomposition caused by residue inputs) especially in soils with high levels of SOC or high C:N ratios [23]. Increases in C mineralization caused by nitrogen fertilization have been positively related to labile C concentrations [24]. Nitrogen fertilization and its interaction with residue addition can prompt C mineralization or immobilization depending on soil type [25], [26]. Greater understanding of how soil physicochemical properties influence soil response to C and N additions is needed to help us manage the soil C cycle to mitigate climate change [27], [28].

The objective of this study was to improve our understanding of SOC mineralization response to residue and N additions in soils with different inherent and dynamic soil properties. Residue additions were expected to result in C accrual in soils that were more degraded or further away from C saturation. Nitrogen additions were expected to increase C mineralization in soils with higher levels of labile C and this effect was expected to be more pronounced in soils with longer rotations assuming that they are better able to increase labile C stocks..

## Materials and Methods

### Soil samples and sites description

Soil samples were collected from two long-term agricultural research sites, one located in central Iowa (IA) and the other in central Maryland (MD). Both studies included cropping system treatments that differ in C input diversity and N fertility sources. The Marsden Farm Cropping Systems Experiment was initiated in 2002 and is located at the Iowa State University Marsden Farm, in Boone County, IA (42°01′ N; 93°47′ W). Average annual precipitation since 1981 is 844 mm and temperature is 9.1°C. Before initiation of the experiment, the IA site had been managed for at least 20 yr with a corn-soybean rotation receiving conventional fertilizer and herbicide inputs. Soils vary across the experimental site and are predominantly Clarion loam (fine-loamy, mixed, superactive, mesic, Typic Hapludolls, 2%–5% slope), Nicollet loam (fine-loamy, mixed, superactive, mesic, Aquic Hapludolls, 1–3% slope) and Webster silty clay loam (fine-loamy, mixed, superactive, mesic, Typic Endoaquolls, 0–2% slope), with smaller areas of Harps loam (fine-loamy, mixed, superactive, mesic Typic Calciaquolls, 0–2% slope), and Canisteo silty clay loam (fine-loamy, mixed, superactive, calcareous, mesic Typic Endoaquolls, 0–2% slope). The experiment compared a conventionally managed 2-yr rotation with 3-yr and 4-yr rotations that are diversified farming systems. Details about rotations, tillage and fertilization are given by Liebman [29] and Davis [30] and summarized briefly in Table 1. The experiment was a randomized complete block design with each crop phase of each rotation system present every year in four replicate blocks. Plot size was 18 m by 85 m.

**Table 1:** Summary of the management, inherent and dynamic soil properties at Marsden and the Farming Systems Project.

	Iowa (Marsden)				Maryland (Farming Systems Project)			
	Site mean	Conv	Med	Long	Site mean	Conv	Med	Long
Crop sequence†		C-S	C-S-oat/rc	C-S-oat/A-A		C-r/S-W/S	hv/C-r/S-W	C-r/S-W/A-A-A
Tillage‡		Ch	Ch, MB	Ch, MB		Ch	D, Ch or MB	D, Ch or MB
Fertilizer sources∮		N	GM, AM, N	GM, AM, N		N, P, K	GM, AM, K	GM, AM, K
Sand (%)	35.2±3.6 A	35.4±4.0 a	35.3±4.4 a	34.8±3.7 a	20.6±6.7 B	23.6±11.6 a	19.0±2.2 a	19.3±3.5 a
Silt (%)	38.5±2.7 B	38.5±4.1 a	38.5±2.3 a	38.4±2.4 a	59.3±5.3 A	55.9±7.4 a	59.5±4.7 a	62.4±1.4 a
Clay (%)	26.4±2.3 A	26.2±2.7 a	26.4±2.9 a	26.8±2.2 a	20.1±3.8 B	20.5±4.7 a	21.5±4.4 a	18.4±2.8 a
pH	7.2±0.4 A	6.9±0.4 a	7.3±0.4 a	7.4±0.3 a	6.4±0.3 B	6.3±0.7 a	6.4±0.1 a	6.4±0.1 a
SOC (g kg^−1^)	28.0±5.6 A	26.4±2.8 a	28.0±9.3 a	29.6±4.1 a	14.1±2.0 B	12.2±2.3 a	15.1±1.0 a	14.9±1.3 a
C:N	12.7±0.6 A	12.9±0.7 a	12.5±0.7 a	12.8±0.5 a	10.7±0.7 B	11.3±0.4 a	10.1±0.3 b	10.7±0.8 b
POM-C (g kg^−1^)	2.8±0.9 A	2.4±0.4 a	3.5±1.4 a	2.7±0.3 a	3.6±0.8 A	2.7±0.5 b	3.7±0.6 a	4.4±0.5 a
POM-C:N	15.7±2.1 A	16.3±1.9 a	15.0±1.9 a	15.9±2.8 a	17.7±3.9 A	21.9±4.3 a	15.0±1.5 b	16.3±1.3 b
OPOM-C (g kg^−1^)	2.2±0.5 B	2.0±0.1 a	2.2±0.2 a	2.3±0.8 a	3.2±0.6 A	2.4±0.4 b	3.2±0.2 a	3.8±0.6 a
OPOM-C:N	12.1±0.6 B	12.7±0.2 a	11.5±0.2 b	12.4±0.8 ab	13.7±0.9 A	14.6±0.5 a	12.9±0.3 b	13.6±1.1 ab

Variables include texture, pH and soil organic carbon(SOC), soil carbon to nitrogen ratio (C:N), particulate organic matter-carbon (POM-C) and POM-C:N ratio, occluded-POM carbon (OPOM-C), and POM-C:N ratio. Data in table are means ± standard deviation.

† C-corn, S-soybean, rc-red clover, A-alfalfa, r-rye cover crop, W-wheat, hv-hairy vetch, W/S-wheat followed by double-cropped soybean. Conv in Maryland followed a 2 yr C-W/S rotation from 1996–1999, Long in Maryland followed a 4 yr C-r/S-W/(r+ orchard grass hay) rotation from 1996–1999.

‡ Ch-chisel plow, MB-moldboard plow, D-disk.

∮ N-urea ammonium nitrate, GM-green manure, AM-animal manures, P-triple super phosphate, K-potassium sulfate. For Iowa site, N fertilization rate was 100 kg N ha−1 with side dressing (0–100 kg N ha−1) as needed based on standard soil tests; green manure was red clover (15.7 Mg ha−1, fresh weight basis) for Med system and was second-year alfalfa for Long system; composted beef cattle manure (on average 128 kg N ha−1) was supplied to both Med and Long systems. For Maryland site, the Conv system received on average 160 kg N ha−1 each year; Med and Long systems received green manure (hairy vetch for Med system, and alfalfa for Long system) and cattle manure (on average 150 kg N ha−1) as N sources.

Values not followed by the same upper case letter differ between two sites (Iowa, Maryland), values not followed by the same lower case letter differ among cropping systems (Conv, Med, Long) within each site. Statistical significances were performed at p<0.05.

The Farming Systems Project was located at the western edge of the Atlantic Coastal Plain at the United States Department of Agriculture-Agricultural Research Service (USDA-ARS) Beltsville Agricultural Research Center in Beltsville, MD (39°03′ N, 76°90′ W). The 30-yr average annual precipitation is 1110 mm; rainfall is distributed evenly through the year. Average annual temperature is 12.8°C. The site had been managed as a row crop production field with continuous no-till for at least 11 years before the study was initiated in 1996. The dominant soil types are Christiana (fine, kaolinitic, mesic Typic Paleudults), Matapeake (fine-silty, mixed, semiactive, mesic Typic Hapludults), Keyport (fine, mixed, semiactive, mesic Aquic Hapludults), and Mattapex (fine-silty, mixed, active, mesic Aquic Hapludults) silt loams. The MD site included five cropping systems [16]; we only sampled the conventional tilled treatment and two of the organic cropping systems (Table 1) to cover a gradient of crop and input diversity that echoed the series of comparisons made in the Marsden Farm plots. Farming systems are replicated four times with each crop phase of each rotation system present every year in a split-plot design with system assigned to whole plots and crop rotation entry point assigned to 111 m by 9.1 m subplots.

These two field studies did not involve endangered or protected species. No written permissions or permits were required to secure access to sample the two experimental trials. Both are publically funded research sites established with the purpose of being sampled.

### Incubation set up

Soil samples were taken from plots or subplots before entering the corn planting phase on 9 May, 2011 in Iowa and 3 June, 2011 in Maryland using four 3–4 cm diameter soil cores to a depth of 20 cm. Composite samples representing the separate blocks were air-dried and sieved to 2 mm for use in the incubation study that compared four treatments for each cropping system soil: un-amended control (Control), N-fertilized treatment (N), residue-amended treatment (R) and treatment with both residue and N-fertilizer (RN). Microcosms were established in 800 mL Mason jars containing 40 g soil. Half of the jars containing soil from each cropping system treatment were amended with residues of wheat (hard red spring wheat, *Triticum aestivum* L.) that had been grown in a greenhouse. Both shoots and roots were cleaned and dried at 40°C, then ground to pass a 1 mm sieve. Dry residue, consisting of 38.6% C and 1.3% N, was added at rate of 0.153 g (40 g)^−1^ soil to approximate a typical field return rate of 8580 kg ha^−1^. The soil was wetted by pipetting an appropriate amount of H_2_O (determined gravimetrically) to reach 50% soil water holding capacity (WHC) and pre-incubated at 4°C for 2 days to allow soil moisture to diffuse evenly and to stimulate microbial activity. Soils were then allowed to warm to room temperature and brought to 60% WHC using either H_2_O or (NH_4_)_2_SO_4_ to produce “no N fertilized” and “N fertilized” (170 kg N ha^−1^) treatments, respectively. There are four replications of each treatment, and the study was a completely randomized block design. The incubation lasted for 330 days.

### Soil analysis and statistics

Soil respiration was quantified by periodic sampling from the headspace of jars incubated in the dark at 24°C. Gas samples were collected from the Mason jars every day for the first 3 days, every other day until day 9, every 3 days until day 30, every 10 days for the next two months, and once per month for the last 8 months of the 11 month study. The CO_2_ concentration in the headspace was measured using an LI-800 CO_2_ Gas Hound Analyzer (Model LI-800, LI-COR). Ports (3 mm diameter) in the top of each jar were sealed with Butly rubber stoppers. Jars were re-aerated and moisture was adjusted to 60% WHC based on a weight estimate as needed after headspace samples were analyzed.

Soil texture, pH and particulate organic matter (POM) content of soils were measured prior to establishing the incubation. Soil texture was estimated by the hydrometer method [31] and pH was measured using an Orion pH electrode method after dispersing soil in distilled water at a soil:water ratio of 1∶1. Soil POM was determined by dispersing soil in sodium metaphosphate solution and collecting material >0.53 µm on a sieve [32]. A subset of soil samples were destructively used for organic matter characterization at 6 and 11 months. The aggregate occluded particulate organic matter (OPOM) was determined using the procedure outlined by Yoo and Wander [33]. Briefly, the light residue of a 20 g soil sample was floated in a centrifuge tube using sodium polytungstate (1.6 g cm^−3^; Geoliquids Inc., Chicago, IL), then removed using 1 µm polycarbonate membrane filters (Osmonics Inc., Minnetonka, MN) after being centrifuged at 5000 rpm for 30 min. The remaining material was shaken at 350 oscillations min^−1^ for 60 min with 50 ml deionized water, then OPOM was collected by 53 µm sieving using poly-carbonate mesh (Gilson Co., Columbus, OH). The POM and OPOM were dried at 80°C and their C and N contents were determined by dry combustion with an Elemental Analyzer (Costech 4010, Costech Analytical Technologies Inc. Valencia, CA).

In soil C mineralization studies, the commonly used models to simulate CO_2_ release include first-order kinetics models, hybrid model (a simplified special case of the two-component model) and double exponential model [19], . Pre-analysis showed that highest r square values (r^2^>0.99) were observed when CO_2_ emission data were fit to a double exponential model. Based on this, and reports that the double exponential model can provide an accurate description of C mineralization for incubations of 200 days or longer [19], [36], we used the double exponential model to describe results from our 330-day incubation. Data were fitted using the following model in PROC NLIN (nonlinear regression) in SAS 9.3:
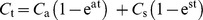
where *C*_t_ is the cumulative amount of CO_2_-C released (g C kg^−1^ soil) in time t; and, *C*_a_ and *C*_s_ represent the active and slow pools of mineralizable C (g C kg^−1^ soil) with decomposition rates of *k*_a_ and *k*_s_ (day^−1^), respectively. Estimates of *C*_a_, *C*_s_ and *k*_a_, *k*_s_ derived from empirically fitting the equation represent predictions about the size and lability of mineralizable fractions. The double exponential model assumes that “resistant carbon pool” (*C*_r_) does not contribute to CO_2_ emissions in a relatively short period [36]. In this study, the *C*_r_ was estimated by subtracting the sum of *C*_a_ and *C*_s_ from total SOC.

The four parameters estimated by nonlinear regression were checked for normality in SAS 9.3 using “univariate normal plot”. The “Mixed” procedure was then performed to quantify the overall effects of site, N fertilization and residue addition (Control, N, R and RN), and their interactions using data from both sites. There was a significant three way interaction between Site, R and N. Associated analysis produced similar information to that supplied by site-based analyses of data. Accordingly, for simplicity’s sake, and to avoid being repetitious, we only report on analyses of sites performed separately. Analyses of our treatments (cropping systems [Conv, Med, Long] and their interactions with N fertilization and residue addition) on measured fractions and estimated coefficients are reported for each site separately because the Conv, Med, and Long cropping systems were not identical at the two locations. Common labeling was used to determine whether generalizations about cropping system effects (eg: rotation length and manure application) might be made. Site-based comparisons were only reported for analyses performed on unamend controls. The multiple comparison of Least Squares Means differences was conducted only when ANOVA results for main factors or factor interactions were significant (Turkey adjusted p<0.05).

## Results and Discussion

### Key site-based differences

**Figure 1:**
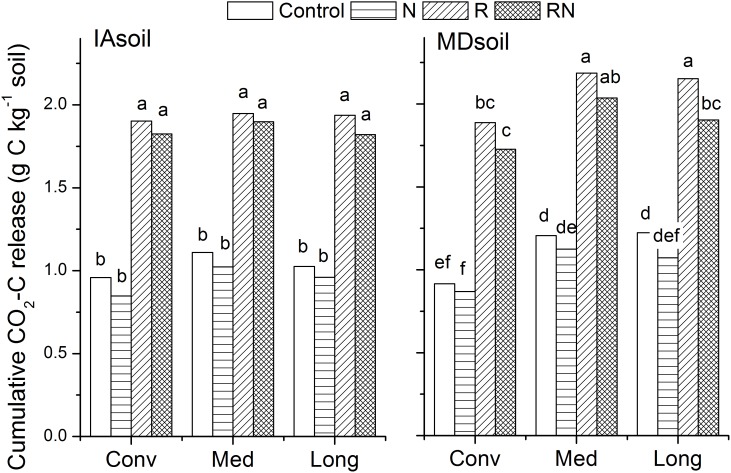
Cumulative CO_2_-C release from the IAsoil and the MDsoil after 330 days incubation. Control: treatments with no residue or N fertilizer added. N: treatments with N fertilization. R: treatments with residue application. RN: treatments applied with both residue and N fertilizer. Different letters above bars suggest significant differences at p<0.05, comparisons were made for the interaction of cropping system (Conv, Med, Long) by treatments (Control, N, R, RN) within each site (IAsoil, MDsoil).

Table 1 summarizes crop management and soil properties measured at each site at the start of the incubation. The Iowa soil (IAsoil) had higher sand and clay contents and pH and a lower silt content than the Maryland soil (MDsoil). The IAsoil also had higher SOC content (28.0 g kg^−1^) and SOM-C:N ratio (12.7) than the MDsoil (SOC = 14.1 g kg^−1^, C:N = 10.7), indicating that organic matter was less decomposed in IAsoil than in MDsoil [22]. The amount of C mineralized from soils was similar at the two sites. Cumulative losses of CO_2_-C ranged from 0.94–1.93 g C kg^−1^ soil in IAsoil, and from 1.02–2.07 g C kg^−1^ soil in MDsoil (Fig. 1). We assumed that temperature and moisture were maintained at or near optimal levels for mineralization [37]–[39] and noted that mineralization rates observed in this study were consistent with soils incubated under similar near-optimal laboratory conditions [40], [41].

When considered as a percentage of SOC, CO_2_-C losses from all treatments in the MDsoil (Control = 7.93, N = 7.26, R = 14.7, RN = 13.2% of SOC) were about twice those from the IAsoil (Control = 3.68, N = 3.33, R = 6.88, RN = 6.59% of SOC). The percentage loss rates from controls are consistent with introductory soils text books [42] that suggest 2%–3.5% of SOC will typically mineralize annually and that larger losses can be expected from soils with less clay. The clay contents of the MDsoil are however, only slightly lower than those of the IAsoil (20% vs 26%). Difference in soil texture, which is commonly used to regulate kinetics in organic matter models as it alters soil water availability, pore size distribution, nutrient availability and surface area [27], does not adequately explain our results. Soil physical protection of SOC is often assumed to be positively related to clay and silt contents [43] but such particle-size based classes poorly reflect critical differences in mineralogy that likely influenced C dynamics in these two soils. The IAsoils are dominated by highly-active smectite clays while the MDsoils are dominated by semi-active kaolinitic clays [44]. By multiplying clay content by typical cation exchange capacity (CEC, smectite = 150–115 cmol_c_ kg^−1^, kaolinite = 0.4–1.5 cmol_c_ kg^−1^) values, we noted that the IAsoil had about 50–100 times more surface activity than the MDsoil [45], [46]. Use of CEC of different clay types to reflect the different types of mineral surfaces that influence surface binding with organic matter [47] rather than clay content to represent differences in inherent soil properties would allow modelers to better estimate soil’s organic C protective capacity. Such an approach could have particular value for efforts seeking to use texture-organic matter associations to target efforts to increase soil C sequestration [48],[49].

In addition to physical protection, modeling efforts assume organic matter quality and quantity control mineralization patterns [50]. Levels of POM, which are often used as an index of SOC status, help predict soil response to amendment. We found that the MDsoil contained a greater percent of SOC as POM-C (25.5%) than did the IAsoil (10.0%) (Table 1), which is consistent with the IAsoil having smaller *C*_a_ and *C*_s_ pools that have slightly larger specific decay rates (*k*_a_, *k*_s_) than the MDsoil (Table 2). By subtracting the sum of *C*_a_ and *C*_s_ from SOC, we estimated that the IAsoil’s protected pool (*C*_r_) was about twice the size of that in the MDsoil. These differences in C pool dynamics are consistent with the notion that the IAsoil afford superior physical protection of SOC by reducing microbial access to SOC [8], [51]–[53].

**Table 2:** Influence of site, management and treatment on active (*C*_a_) and slow (*C*_s_) carbon pool sizes and their decomposition rate constants (*k*_a_, *k*_s_, respectively).

Factors			*C*_a_	*k*_a_	*C*_s_	*k*_s_	*C*_r_
Site	Treatment		(g C kg^−1^ soil)	(day^−1^)	(g C kg^−1^ soil)	(day^−1^)	(g C kg^−1^ soil)
Iowa	Control		0.16±0.03 B	0.16±0.02 A	1.07±0.18 B	0.0047±0.0010 A	25.2±3.9 A
(IAsoil)		Conv	0.14±0.02 b	0.18±0.01 a	1.17±0.15 a	0.0036±0.0008 a	25.1±2.7 a
		Med	0.17±0.01 ab	0.15±0.01 a	1.02±0.14 a	0.0054±0.0005 a	22.2±3.4 a
		Long	0.19±0.02 a	0.16±0.02 a	1.01±0.23 a	0.0051±0.0009 a	28.3±4.2 a
	All trts	Conv	0.43±0.30 a	0.15±0.07 a	1.27±0.26 a	0.0042±0.0007 a	25.0±2.4 a
		Med	0.48±0.32 a	0.13±0.05 a	1.29±0.25 a	0.0051±0.0007 a	22.1±2.6 a
		Long	0.47±0.29 a	0.13±0.05 a	1.19±0.28 a	0.0050±0.0008 a	27.9±4.0 a
		Control, N	0.17±0.03 b	0.19±0.03 a	1.05±0.16 b	0.0046±0.0009 a	25.2±3.7 a
		R, R+N	0.75±0.06 a	0.08±0.01 b	1.45±0.18 a	0.0049±0.0007 a	24.8±4.0 b
		Control, R	0.47±0.31 a	0.12±0.04 b	1.28±0.25 a	0.0049±0.0009 a	24.9±3.8 a
		N, R+N	0.45±0.29 a	0.15±0.06 a	1.22±0.27 a	0.0046±0.0007 a	25.0±3.9 a
Maryland	Control		0.23±0.02 A	0.13±0.02 B	1.29±0.16 A	0.0038±0.0011 B	12.5±1.9 B
(MDsoil)		Conv	0.22±0.02 a	0.11±0.02 a	1.29±0.22 a	0.0024±0.0006 b	10.6±2.0 b
		Med	0.23±0.03 a	0.14±0.02 a	1.35±0.14 a	0.0040±0.0002 a	13.5±0.8 a
		Long	0.23±0.03 a	0.15±0.03 a	1.21±0.09 a	0.0049±0.0007 a	13.4±1.1 a
	All trts	Conv	0.51±0.29 a	0.11±0.01 b	1.33±0.29 a	0.0031±0.0008 c	10.2±1.6 b
		Med	0.53±0.28 a	0.13±0.01 a	1.38±0.23 a	0.0050±0.0010 a	13.3±0.8 a
		Long	0.51±0.28 a	0.12±0.02 ab	1.46±0.20 a	0.0041±0.0009 b	13.0±1.3 a
		Control, N	0.87±0.03 b	0.12±0.02 a	1.26±0.18 b	0.0034±0.0010 b	12.5±1.6 a
		R, RN	2.90±0.05 a	0.11±0.01 b	1.52±0.22 a	0.0047±0.0010 a	11.8±1.9 b
		Control, R	1.92±0.30 a	0.12±0.02 a	1.42±0.20 a	0.0044±0.0012 a	12.1±1.9 a
		N, RN	1.86±0.26 a	0.12±0.01 a	1.36±0.28 a	0.0037±0.0010 b	12.2±1.7 a

All coefficients were estimated by modeling Ct = Ca (1-e^–ka t^)+ Cs (1-e^–ks t^) using cumulative CO_2_ emission data. Results are means ± standard deviation for unamend control soils (Control) and all treatments (All trts).

Control: treatments with no residue or N fertilizer added. N: treatments with N fertilization. R: treatments with residue application. RN: treatments applied with both residue and N fertilizer. “Control, N” and “R, RN” represent the treatments without or with residue addition respectively; “Control, R” and “N, RN” represent the treatments without or with N fertilization respectively.

Grand mean comparisons between sites were made with controls, values not followed by the same upper case letter differ at p<0.05. Means comparisons within each site were made within treatment groups, i.e. cropping system (Conv, Med, Long), residue addition (“Control, N”, “R, RN”), N fertilization (“Control, R”, “N, RN”), values not followed by the same lower case letter differ at p<0.05.

**Figure 2:**
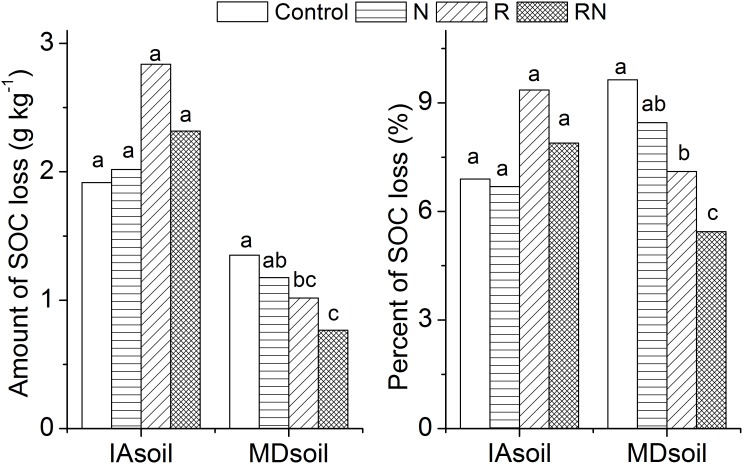
Amount and percentage of SOC loss comparing with initial SOC content. Control: treatments with no residue or N fertilizer added. N: treatments with N fertilization. R: treatments with residue application. RN: treatments applied with both residue and N fertilizer. Different letters above the bar suggest significant differences at p<0.05, comparisons were performed in different treatments within each site (IAsoil, MDsoil).

Those results are also consistent with the greater percentage of SOC loss as CO_2_ from the MDsoil but at odds with directly measured losses in SOC, which were greater from the IAsoil (1.91–2.84 g kg^−1^) than from the MDsoil (0.76–1.35 g kg^−1^) (p<0.01) (Fig. 2). Note, however, that the percentage of SOC loss relative to initial SOC contents was similar for the two sites (6.6%–9.4% in the IAsoil vs 5.4%–9.7% in the MDsoil) (Fig. 2). Losses in SOC were greater than could be accounted for by CO_2_-mineralization in the IAsoil and in the Control treatment of MDsoil; SOC must have been lost to dissolved organic carbon (DOC) or in volatile forms including CH_4_ that were not measured during the incubation. Work by Bellamy [54] suggesting DOC losses are greater from high active soils like the IAsoil supports this notion. Observed losses of SOC are consistent with the faster decay rates (*k*_a_ and *k*_s_) observed for IAsoil. More rapid decay in the IAsoil might be due to the porosity which allowed those soils to maintain more water at 60% WHC (moisture = 35.7% in IAsoil vs 29.6% in MDsoil).

Efforts to predict soils’ response to management often focus more on labile fractions than on resistant fractions. Models of C dynamics commonly assume that about half of the C contained in residues or active fractions will be mineralized annually [55]. Direct measurement of particulate organic matter has been proposed as one way to estimate mineralization [10]. The observed CO_2 _mineralization from un-amended controls (IAsoil = 0.99 g C kg^−1^ soil and MDsoil = 1.07 g C kg^−1^ soil) (Fig. 1) were about one third that contained in POM-C (IAsoil = 2.8 g C kg^−1^ soil and MDsoil = 3.6 g C kg^−1^ soil) (Table 1) at the start of the incubation. Differences in the size of estimated labile pools parallel differences in POM-C and OPOM-C observed in the two sites; this suggests that these direct measures can provide useful estimates of C vulnerability to decay [52]. Changes in OPOM-C contents observed over the course of the incubation were positively correlated to net SOC loss (IAsoil: r^2^ = 0.40, n = 48, p<0.05; MDsoil: r^2^ = 0.38, n = 48, p<0.05). Similarly, total POM-C contents were also correlated with SOC loss (IAsoil: r^2^ = 0.45, n = 48, p<0.01; MDsoil: r^2^ = 0.45, n = 48, p<0.01). However, no correlation was found between initial OPOM-C contents and *C*_s_ size, and OPOM-C net loss during incubation was negatively related to estimates of *C*_s_ in both soils (IAsoil: r^2^ = −0.35, n = 35, p<0.05; MDsoil: r^2^ = −0.31, n = 46, p<0.05), and to *C*_a_ in the MDsoil (r^2^ = −0.74, n = 46, p<0.01). Negative correlations suggest organic matter fractions other than OPOM contribute to mineralization.

### Cropping Systems

Use of diversified cropping along with organic fertilizers established greater differences in dynamic soil properties in the MDsoil than the IAsoil. Cropping system-based differences in IAsoil’s dynamic properties were not statistically significant in this study (Table 1) due to high variability among a small number of samples. The POM-C patterns in IAsoil (Med≥Long≥Conv) agree with recent work [56] using more extensive sampling from two soil depth (0–10 cm, 10–20 cm). That recent work showed the POM-C contents in the IAsoil were significantly higher in the Med and Long systems than the Conv system, which were principally due to increases in POM-C and potentially mineralizable nitrogen in the 10–20 cm depth of soils in the diversified rotations. Our study considered the 0–20 cm depth as a whole, and sampled only plots about to be planted with corn. Variability among IAsoil from different blocks, which were kept separate in 4 replications, prevented us from finding significant differences of POM-C fractions among systems; but the OPOM-C:N ratio was lower in the Med than the Conv system (p<0.05). In the MDsoil, longer cropping systems with more diverse C input (poultry litter and legume cover crop inputs) had significantly increased POM-C and OPOM-C contents (Long≥Med>Conv), and decreased C:N ratios of SOM, POM (Med≤Long<Conv) and OPOM (Med<Conv) (Table 1). These findings are consistent with Spargo [16] who reported that organic cropping systems increased POM-C contents and decreased POM-C:N ratios in the MDsoil.

**Figure 3:**
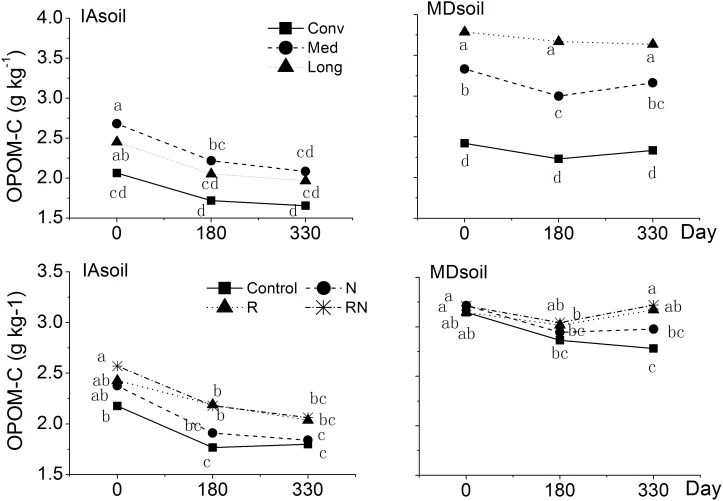
Effect of cropping system and treatment on occluded POM-C (OPOM-C) dynamics during 330 day incubation. Control: treatments with no residue nor N fertilizer added. N: treatments with N fertilized, R: treatments with residue applied. RN: treatments with both residue and N fertilizer added. Different letters suggest significant differences at p<0.05, comparisons were were made for the interaction of cropping system (Conv, Med, Long) by date (0, 180, 330 days), and treatments (Control, N, R, RN) by date within each site (IAsoil, MDsoil).

The more notable influence of cropping system on soil C pools observed in the MDsoil was expressed in cumulative CO_2_ emissions; losses of CO_2_-C (1.61 g C kg^−1^ soil) from Med and Long were greater than from the Conv treatment (1.30 g C·kg^−1^ soil) (p<0.05) (Fig. 1). Cumulative CO2 emissions did not vary among systems in the IAsoil. Use of diversified rotations in IAsoil did increase the *C*_a_ pool size in control soils (Long≥Med≥Conv) but only differences between the Long and Conv were significant; while for the MDsoil controls, both *k*_s_ and *C*_r_ were significantly greater in the Long and Med than in the Conv system (p<0.05) (Table 2). For both sites, greater OPOM-C values were observed in soils from Med than from Conv systems, and OPOM-C stocks declined significantly in the Med systems in both soils and Long system in the IAsoil during 0–180 days of the incubation (p<0.05). During the last 180–330 days, the OPOM stocks remained unchanged in all systems in the IAsoil and increased slightly in the Med system in the MDsoil (p = 0.0507) (Fig. 3).

By removing loose-POM, which contains organic residues and roots that are not well decomposed or bound to minerals, we resolved differences among cropping systems treatments that were obscured by the presence of the more labile residues, suggesting stronger predictive power of OPOM than total POM. We had expected differences of *C*_a_ and *C*_s_ among systems to parallel those observed for POM-C and OPOM-C but cropping system had little influence on these estimated coefficients (Table 2). Here both the directly measured fractions and coefficients derived from incubations suggest that C dynamics in the MDsoil are more strongly influenced by management and that aggregate protection plays a more important role. Recent work [56] asserted decay rates in the IAsoil must vary among systems to maintain similar levels of SOC despite differences in C input levels. The absence of differences in *C*_r_ pool size and modest differences of labile fractions observed among cropping systems (Table 2) in the IAsoil are consistent with the characteristics [57] assigned to C saturated soils. The faster decay rates observed in IAsoil (Table 2) are also consistent with the notion that SOC stabilization capacity could be reduced in C saturated soils [11], [58]. Carbon saturation of the IAsoil might help explain why net SOC losses might be derived from stocks attributed to the resistant pool.

### Residue and Nitrogen Additions

Wheat residue addition doubled the amount of C mineralized to CO_2_ at both sites (Fig. 1) presumably by increasing the activity, size and possibly the composition of the microbial community [59], [60]. The CO_2_ loss ranked R = RN>Control = N. Shifts in the microbial community resulting from changes in C availability likely occurred during the incubation, shifting from those specializing in the decay of fresh organic-materials (*r*-strategists), to *K*-strategists that can consume soil native SOM [59], [61], [62] but those changes would not be clearly revealed by our analyses. Factors regulating CO_2_ loss from this type of ‘curve fitting’ study are typically assumed to include organic matter quality and quantity and soil’s physical protection while assuming that microbial community acts as a catalyst [50]. Accordingly, soil amendment will only change pool sizes and rate coefficients with changes in microbial activity being reflected in the rate coefficient.

Residue addition increased *C*_a_ by 340% and *C*_s_ by 38% in the IAsoil (p<0.05). For the MDsoil, residue addition increased *C*_a_ by 230%, *C*_s_ by 21% and *k*_s_ by 38% (p<0.05) (Table 2). Ladd et al. [63] found decay rates were reduced more by residue addition in soils with reactive minerals than in lower activity soils, particularly during the early phases of decay. This is consistent with our findings, where *k*_a_ declined by 58% in IAsoil compared to just 9.1% in MDsoil after residue addition (Table 2). In the IAsoil, residue additions altered *k*_a_ more than *k*_s_, suggesting that the *C*_s_ pool were less affected by amendment. Even though residue addition increased the size of both *C*_a_ and *C*_s_ in both sites, reductions in *C*_r_ in the MDsoil suggest amendment primed losses of SOC from the resistant pool. Residue additions slightly but non-significantly increased the amount and percentage of SOC loss from IAsoil but significantly reduced both the amount and percentage of SOC loss compared to the Control and N treatments in MDsoil (p<0.05) (Fig. 2). These opposite responses to residue amendment might suggest that there was more labile C vulnerable to priming effects present in IAsoil than in MDsoil [23]. This notion is consistent with the higher C:N ratios observed in IAsoil but does not agree with estimates of labile reserves derived from curve fitting that suggest the IAsoil stocks (*C*_a_+*C*_s_) are smaller than those present in the MDsoil. The notion that *C*_r_ contributes to decay in the IAsoil due to C saturation could explain this discrepancy. Neither N fertilization nor residue application increased OPOM-C (p>0.05). However, trends in OPOM-C, which decreased throughout the incubation in IAsoil, reinforce the notion that that soil has limited capacity to absorb additional C. In the MDsoil, residue addition caused OPOM-C stocks to increase during the later phase of the incubation (Fig. 3), suggesting that the MDsoil was better able to stabilize additional C and that aggregate protection of labile C played an important role at that site.

Nitrogen fertilization consistently but non-significantly reduced the amount of CO_2_-C loss from both soils (Fig. 1). Reduction of C mineralization caused by N fertilization is likely due to two reasons: 1) the increased microbial growth resulting from a reduction of soil C:N ratios from 10.1–13.7 (C:N ratio of residue used is 30) to values closer to that of soil microbes (4.1–12.1) [64] following fertilization, and 2) soil microbes with N addition have a lower requirement for organic N sources and thus the amount of organic matter mineralization needed to support microbial growth was reduced. Nitrogen fertilization increased *k*_a_ of IAsoil by 25% and decreased *k*_s_ of MDsoil by 16% (p<0.05) (Table 2). These differences again seem to be associated with differences in SOM saturation and protective capacity of the two soils.

**Figure 4:**
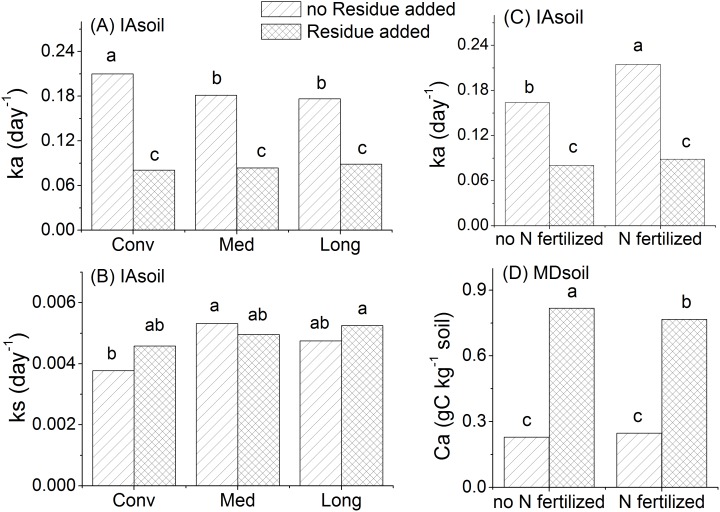
Interactions between residue addition and cropping system or N fertilization on C mineralization coefficients. Different letters above bars indicate statistically significant difference at p<0.05, comparisons were made within each sub-figure.

Residue additions interacted with cropping system and/or with N additions in both soils (Fig. 4). In the IAsoil, residue addition reduced *k*_a_ by 58%, and removed differences in *k*_a_ observed among controls with different cropping histories where *k*_a_ was greater in Conv than Med and Long (Fig. 4A). Residue addition also alleviated differences in the *k*_s_ observed for controls (Med>Conv) from different cropping systems (Fig. 4B). When residue and N fertilizer were applied together, N addition increased *k*_a_ values of the IAsoil by 31% over the control when no residue was applied but *k*_a_ remained unchanged after N addition when residue was added (Fig. 4C). There was also a significant interaction between residue addition and N fertilization on *C*_a_ in the MDsoil, where residue addition prompted a smaller (2.1 fold) increase in *C*_a_ in N fertilized soils and a 2.6 fold increase in unfertilized soils (Fig. 4D).

## Conclusion

This work showed that by using CEC or surface charge, rather than texture or clay content, to represent inherent differences in soil protective capacity, we were able to account for differences in C mineralization response to residue and nitrogen fertilization observed in different soil types. By using dynamic soil properties (POM, OPOM) we were able to anticipate important differences in C dynamics observed in the soils from different sites. More diverse cropping notably improved soil dynamic properties and accelerated C mineralization in the MDsoil. Use of diversified rotations produced more limited effects on dynamic properties in the IAsoil. Residue application increased the size of *C*_a_ and decreased *k*_a_ in both soils, but the effect was more pronounced in the IAsoil than the MDsoil. As predicted, residue addition reduced SOC loss and promoted C immobilization in OPOM in the MDsoil where SOC levels were low. The effect of residue addition on SOC loss from the IAsoil, which appears to be at or near C saturation levels, is consistent with the observations of Zhang [23] that linked high SOC to priming. The greater vulnerability of the IAsoil to C priming was not associated with POM-C levels, which were low compared to those observed in the MDsoil, but was associated with higher SOM-C:N ratios and loss from the resistant pool. Our results suggest that the proportion of SOC in POM-C does not reveal soil C saturation status in a way that can be generalized or be used on its own to predict the ability of soils to sequester or lose SOC. Use of OPOM, rather than POM or C mineralization fitted parameters, to evaluate protection by aggregates, helped resolve important differences in C cycling within the two soils. Nitrogen fertilization did not induce priming (net SOC loss or CO_2_ loss) from either soil; in fact, additions suppressed net C mineralization from both soils. Nitrogen addition caused increased *k*_a_ in the IA soil and in *C*_s_ in the MDsoil when added with residues; this indicates N might have limited early stages of decay in the IAsoil and C assimilation in the MDsoil. Future research should explore potential for use of CEC and POM to initialize, or adjust coefficients used within carbon cycle process models.
